# Design and Characterization of an Osmotic Pump System for Optimal Feeding and pH Control in *E. coli* Culture to Increase Biomass

**DOI:** 10.5812/ijpr-138677

**Published:** 2024-02-17

**Authors:** Saeedeh Abedin, Javad Ranjbari, Azadeh Haeri, Hossein Vahidi, Hamid Reza Moghimi

**Affiliations:** 1 Department of Pharmaceutics and Pharmaceutical Nanotechnology, School of Pharmacy, Shahid Beheshti University of Medical Sciences, Tehran, Iran; 2 Department of Biotechnology, School of Advanced Technologies in Medicine, Shahid Beheshti University of Medical Sciences, Tehran, Iran

**Keywords:** Osmotic Pump, Batch Culture, Glucose, Sodium Carbonate, *E. coli*, Controlled Release

## Abstract

**Background:**

Batch cultures used for various purposes, such as expression screening and recombinant protein production in laboratories, usually have some drawbacks due to the bolus addition of carbon sources, such as glucose and buffers, that lead to overflow metabolism, decreased pH, high osmolality, low biomass yield, and low protein production.

**Objectives:**

This study aimed to overcome the problems of batch culture using the controlled release concept by a controlled porosity osmotic pump (CPOP) system.

**Methods:**

The CPOP was formulated with glucose as a carbon source feeding and sodium carbonate as a pH modifier in the core of the tablet that was coated with a semipermeable membrane containing cellulose acetate and polyethylene glycol (PEG) 400. The release rate was regulated with Eudragit L100 as a retardant agent in the core and PEG 400 as a pore-former agent in the coating membrane. Fourier-transform infrared spectroscopy (FT-IR) and scanning electron microscopy (SEM) were used to elucidate compatibility between components and release mechanism, respectively. The in-vitro release of glucose and Na_2_CO_3_ studies were performed for 24 hours in a mineral culture medium (M9). Then, the effectiveness of CPOP in the growth of *Escherichia coli *(*E. coli* *BL21*) as a microorganism model was evaluated. Glucose consumption, changes in medium's pH, and acetate concentration as a by-product were also monitored during the bacterial growth.

**Results:**

Fourier-transform infrared spectroscopy confirmed the compatibility between the components in the osmotic pump, and SEM elucidated the release mechanism due to in-situ delivery pores created by dissolving soluble components (PEG 400) on the coated membrane upon contact with the dissolution medium. The in-vitro release studies indicated that the osmotic pump was able to deliver glucose and sodium carbonate in a zero-order manner. The use of CPOP in *E. coli* (BL21) cultivation resulted in a statistically significant improvement in biomass (over 80%), maintaining the pH of the medium (above 6.8) during the exponential phase, and reducing metabolic by-product formation (acetate), compared to bolus feeding (P < 0.05).

**Conclusions:**

The use of CPOP, which is capable of controlled release of glucose as a carbon source and sodium carbonate as a pH modifier, can overcome the drawbacks of bolus feeding, such as decreased pH, increased acetate concentration, and low productivity. It has a good potential for commercialization.

## 1. Background

Recombinant proteins are one of the fastest-growing research fields in many industries, such as pharmaceutical, food, and cosmetic products (1, 2). The industrial strategy for the production of recombinant proteins is fed-batch cultivation with the controlled addition of substrates to culture and automated pH monitoring; however, on a laboratory scale, the production is carried out in shake flasks in the batch operational mode for screening production strains and process development (3). However, there are some limitations associated with batch culture in flasks, such as transiently elevated concentration of nutrients and metabolites, loss of pH control, and low aeration that result in a decreased culture density and might mislead the researcher from choosing the most appropriate strain and designing process experiments (4). 

The bolus addition of feeding substrates or buffers at the outset of processes might lead to high osmolality and decrease the growth rate of microorganisms (5). Furthermore, high initial substrate concentrations potentially cause rapid growth of microorganisms and oxygen deficiency, consequently the secretion of large amounts of growth-inhibiting by-products, such as formic, acetic, and lactic acids and ethanol that might lead to a pH drift; nevertheless, there is a specific optimal pH range for the best growth of each microorganism (6, 7). 

To overcome these problems, many efforts have been put into the application of the fed-batch strategy to improve biomass density in shake flasks, one of which is employing controlled release systems and devices. For example, extended-release systems for releasing glucose or sodium carbonate were designed using silicon matrices (8, 9). Glucose- or magnesium hydroxide-loaded hydrogels are also reported for the sustained release of nutrients and pH maintenance to improve biomass yields (10, 11).

Another approach is enzyme-based controlled release systems based on enzymatic reactions to break down starch into glucose, which is commercially available as ready­made media, the EnBase^®^ and EnPresso^®^ B systems (12, 13). Enzyme-based controlled release systems are sensitive to enzymes and enzyme inhibitors in culture medium and also to other conditions such as the pH and temperature of the medium (3). 

An alternative novel controlled delivery system that has the potential to feed nutrients in a controlled manner and control pH in a shake flask is the osmotic pump formulation, the subject of the present investigation. An osmotic pump is composed of an inner core containing active ingredients and osmogens coated with a semipermeable membrane. During operation, the core absorbs water and pushes the drug solution out through the delivery pores (orifice) (14). The rate of cargo release in osmotic pumps is usually independent of the pH and hydrodynamic condition of the medium, and a zero-order drug release profile can be obtained after an initial lag time (15). 

Among different types of osmotic pumps, the controlled porosity osmotic pump (CPOP) model was chosen to circumvent the creation of an orifice either by laser drilling or other techniques on account of the creation of in-situ microporous membranes after dissolving water-soluble additives in the coating membrane upon contact with an aqueous environment (16, 17). In the development of the core tablet of the osmotic pump, glucose was used as the preferred carbon source feeding (18, 19). Meanwhile, due to its osmogenic properties, which create a driving force to imbibe water inside the tablet (14). Sodium carbonate was used to modify the pH of the medium during *Escherichia coli* (*E. coli*) growth. The core tablet was covered with a semipermeable membrane comprising cellulose acetate (CA) as a water-insoluble polymer and polyethylene glycol 400 (PEG 400) as a water-soluble pore-forming agent. The osmotic pump was then used in the cultivation of *E. coli*, the most popular host microorganism in biotechnology (20), to improve bacterial growth.

## 2. Methods

### 2.1. Materials

D-glucose monohydrate was purchased from Bio Basic (Toronto, Canada). Sodium carbonate (Na_2_CO_3_) was provided from Panreac (Barcelona, Spain). Eudragit L100 was obtained from Evonik Rohm Pharma GmbH (Essen, Germany). Cellulose acetate (CA) (MW: 30000), 3,5 dinitrosalicylic acid (DNS), sodium potassium tartrate (Rochelle salt), microcrystalline cellulose (MCC), polyvinylpyrrolidone (PVP) K-30, acetone, and PEG 400 were provided by Merck/Sigma-Aldrich (Germany). All of the solvents and chemicals were of analytical grade. 

### 2.2. Microorganisms and Cultivation Media

*Escherichia coli BL21* (*DE3*) was used as a model microorganism. Pre-culture was performed in LB medium at 37°C overnight. The *E. coli* main cultivation for the experiment was performed in a mineral M9 medium that was purchased from Biobasic (Toronto, Canada). Details are explained in the Appendix 1 (in the Supplementary File). 

### 2.3. Analytical Methods

Analytical methods for evaluating cell growth (optical density at 600 nm, (OD_600_), pH, and carbonate release are described in Appendix 1 (in the Supplementary File). Acetate was measured by a K-acetrm (Megazyme, Ireland) kit based on acetate kinase and phosphotransacetylase according to its manual. Glucose was assayed spectrophotometrically with DNS method (21, 22) based on the reduction reaction between glucose and DNS, with details mentioned in Appendix 1 (in the Supplementary File).

### 2.4. Preparation of Controlled Porosity Osmotic Pumps

The osmotic pumps were prepared by coating the tablet using in-situ pore formers as follows:

#### 2.4.1. Tablets Core 

Different core tablets were prepared using a wet granulation technique using components listed in Table 1. Na_2_CO_3_, Eudragit L100, and half of the glucose were granulated with an alcoholic solution of PVP K-30. Thereafter, the other half of the glucose and MCC were also granulated separately. Granules were dried in a drying oven at 40°C and were then passed through sieve No. 80 (US standard size, 177 µm). Magnesium stearate was added to them as the lubricant, and the resultants were compressed into round tablets with a standard single punch press tablet machine (Erweka AR 400, Germany) equipped with a 20-mm round die. 

#### 2.4.2. Tablets Coat

The coating solution was prepared by dissolving CA (4% w/v) in acetone/alcohol (90:10, v/v). Then, PEG 400 was added into the solution as a pore-forming agent and a plasticizer at CA: PEG 400 ratios of 2:0.5, 2:1, 2:1.5 (all w/w). A dip-coating technique was employed for coating (20, 21), and the process was repeated until reaching a 10% increase in the initial weight of tablets to ensure complete coverage.

**Table 1.** Formulation Components of Different Controlled Porosity Osmotic Pumps Containing 1000 mg Glucose, 40 mg Eudragit L100, 25 mg Magnesium Stearate, and Different Amounts of Microcrystalline Cellulose (MCC) and Na_2_CO_3 _in the Core Formulation and Different Amounts of Cellulose Acetate (CA) and Polyethylene Glycol (PEG) 400 in Coat Solution

Formulation Code	Core (mg)		Coat
	Na_2_CO_3_	MCC	CA: PEG 400
**F1**	200	235	2: 1
**F2**	400	35	2: 1
**F3**	400	35	2: 1.5
**F4**	400	35	2: 0.5

Abbreviations: MCC, microcrystalline cellulose; CA, cellulose acetate; PEG, polyethylene glycol.

### 2.5. Characterization of Osmotic Pumps 

#### 2.5.1. Uniformity of Dosage Unit and Hardness

Twenty tablets of each formulation were weighed individually, and the weight variation test was performed according to the United States Pharmacopeial Convention (USP) criteria (23). The hardness of 10 core tablets was determined using a hardness tester (Erweka TBS 325, GmbH) before coating. 

#### 2.5.2. Fourier-Transform Infrared Spectroscopy

To study the compatibility of ingredients, Fourier-transform infrared spectroscopy (FT-IR) (IFS 55; Bruker Corp, Billerica, MA, USA) was performed over the range 4000 - 650 cm^-1^ on samples of glucose, sodium carbonate, and core tablet powder, using the potassium bromide (KBr) pellet method.

#### 2.5.3. Scanning Electron Microscopy

A scanning electron microscope (SEM) (Sigma VP, ZEISS, Germany) was employed to observe the morphology and microstructure of semipermeable membranes before and after 24 hours of contact with the M9 medium and release study.

#### 2.5.4. In-vitro Release Studies

In-vitro release studies of the osmotic pump tablets were conducted at conditions simulating bacterial cultures at 37 ± 0.5°C under shaking at 180 rpm. The tablets were added to 100 mL M9 medium culture with 0.1% sodium azide (to avoid contamination). Glucose concentration was determined by the DNS method. The amount of sodium carbonate released was measured by determining pH at each sampling time and using a calibration curve.

To compare *in-vitro* release profiles of different formulations, the similarity factor (f_2_) (Equation 1) was calculated (19, 20). The similarity was confirmed when the calculated f_2_ value was higher than 50.

Equation 1.


$$f_{2}=50\times log\{[1+\frac{1}{n}{\sum_{t=1}^{n}{\;\left(R_{t}-T_{t}\right)}^{2}]}^{-0.5}\times 100$$

where R_t_ and Tt are the cumulative releases of reference and test formulations at each time point, respectively, and n is the number of time points.

#### 2.5.5. Release Kinetics Determination 

The data obtained from the release of glucose and sodium carbonate were fitted into different mathematical Equations (2-4) to predict the kinetic release of the tablets (24) as follows:


$$\;{Zero\;order:Q}_{t}\;=\;k_{0}t$$

$$\;First\;order:ln\;Q_{t}\;=\;lnQ_{0}\;\;–k_{1}t$$

$$Higuchi:Q\;=k_{H}{\;t}^{1/2}$$

where Q is the cumulative percentage of drug released in time t, and k is the release constant related to each mathematical kinetic model. The highest fit (r^2^) was taken as a criterion for selecting the most appropriate model.

### 2.6. Efficacy of the Osmotic Pump Measured by E. coli Growth

The effects of osmotic pumps, with or without bolus glucose (4 g/L), compared to control (no tablet, glucose 4 or 12 g/L), on the growth and stabilization of pH in *E. coli* culture in M9 medium were investigated. *Escherichia coli* was chosen due to its easy application, rapid growth, and cheap host for recombinant protein expression (25). 

Tablets were first sterilized by swabbing their surface with ethanol (70%) and then ultraviolet (UV) light for 15 minutes on each side of the tablets in aseptic laminar airflow. The ability of this method for sterility of CPOP was confirmed through a direct transfer sterility testing method (26) by incubating the sterile CPOP in two autoclaved mediums, thioglycolate (FTM) at 37°C, and soybean casein digest medium (SCDM) at 25°C for 14 days followed by the inspection of the media for microbial or fungal contamination. 

One tablet was added to a 100 mL M9 culture medium that was inoculated with *E. coli* from an overnight pre-culture (10: 1) in a shaking flask. The test was performed at 37°C and 180 rpm for 24 hours. No tablets were used in the control flask. Samples of all the culture flasks were taken at regular intervals for the measurement of OD (growth), pH, glucose, and acetate concentration.

### 2.7. Statistical Analysis 

The Student’s *t*-test was used to compare the release data of different formulations. A one-way analysis of variance test (ANOVA) was conducted to investigate whether there was any difference in bacterial growth, pH of the culture medium, or acetate production using designed formulations. P-values of < 0.05 were considered statistically significant.

## 3. Results

### 3.1. Characterization of Control Porosity Osmotic Pump 

#### 3.1.1. Physical Evaluation 

Weight variation tests were performed to confirm the uniformity of dosage units in prepared formulations. Core tablet weights varied between 1483.2 mg and 1500.5 mg (mean 1485.2 mg); nevertheless, the weight of none of the 20 tablets showed more than 2% deviations from the average weight in agreements of USP acceptance criteria (23). The thickness of the core tablets was in the range of 3.65 to 3.74 mm (mean 3.70 mm). The hardness of core tablets was found to be between 8.56 and 11.93 kg cm^–2^ (mean 10.24 kg cm^–2^). Therefore, the weight variation and physical parameters of different formulations fulfilled the acceptance practical criteria. 

#### 3.1.2. Drug-Excipient Compatibility by FT-IR

**Figure 1.**
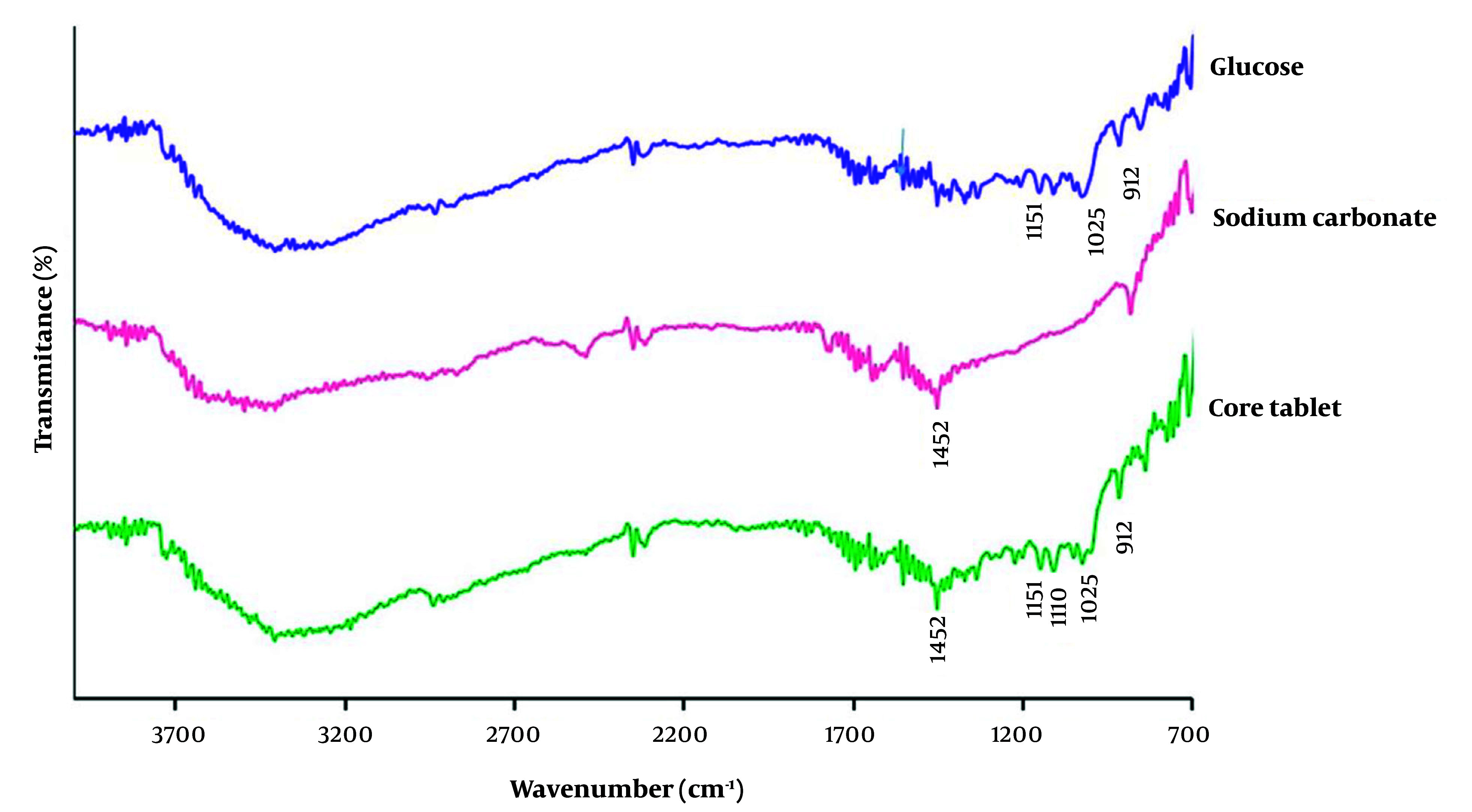
FT-IR spectra of the pure glucose, pure sodium carbonate, and the core tablet of the osmotic pump show no interaction among the ingredients.

Figure 1 depicts the FT-IR spectra of pure glucose, sodium carbonate, and the core tablet. In the FT-IR spectrum of glucose, the characteristic sharp peaks are shown at 1111 cm^-1^ due to CO stretching vibration, at 912 cm^-1^ corresponding to vibrations of CO and CCH of pyranose ring and 1025 cm^-1^ and 1151 cm^-1^ corresponding to primary alcohol and secondary alcohol, respectively (27, 28). Additionally, the characteristic sharp peak of sodium carbonate as a pure powder located at 1453 cm^-1^ is assigned to the stretching vibration of CO_3_^2 ^(29). The peaks observed in the FT-IR spectra of pure drugs were found in the FT-IR spectra of the core tablets. Therefore, the results showed no chemical interaction among the tablet components.

#### 3.1.3. Scanning Electron Microscopy of Microporous Membrane Structure

**Figure 2.**
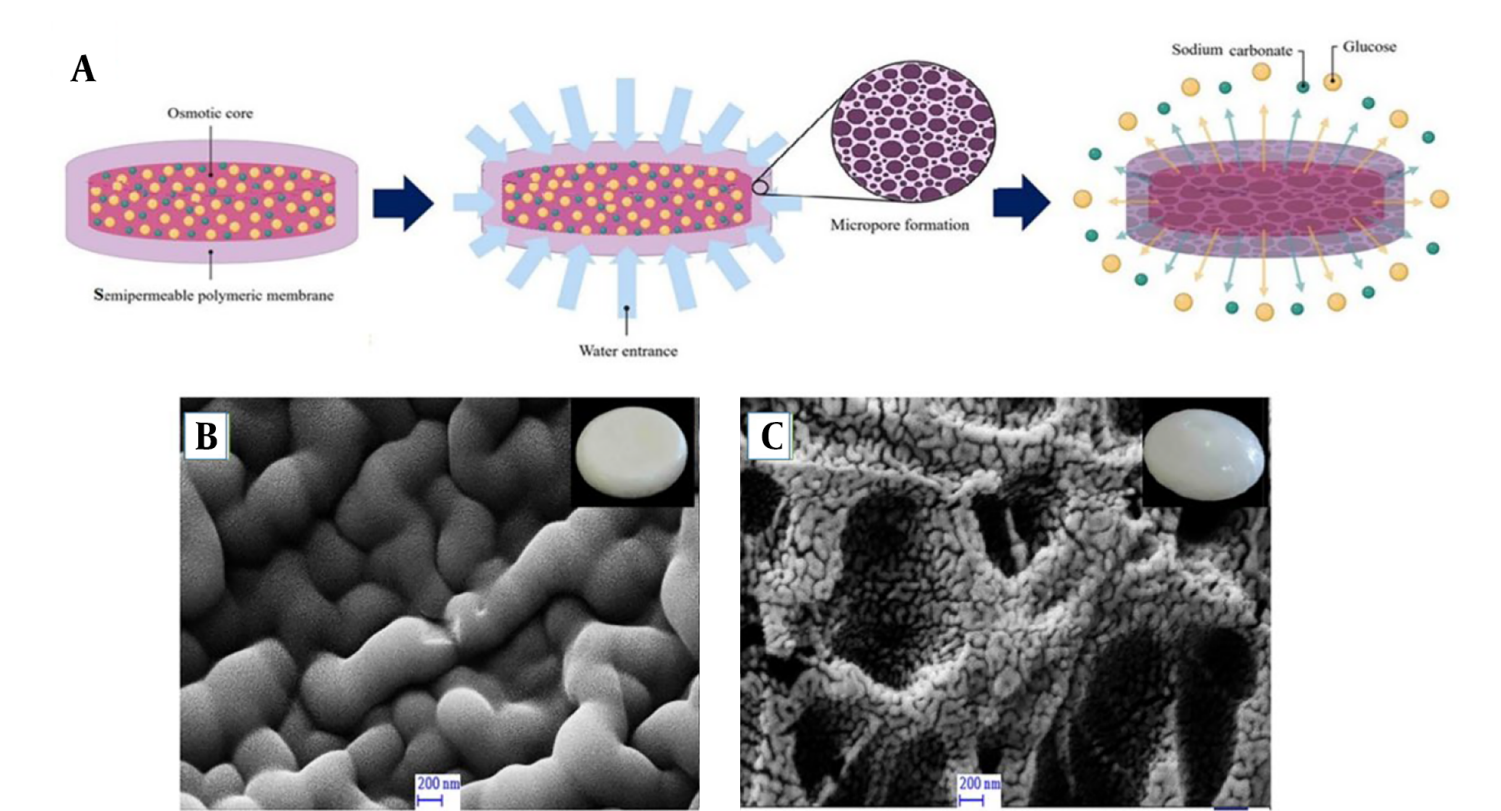
A,Schematic diagram of the release mechanism of controlled porosity osmotic pump, showing pore formation upon leaching the pore former (PEG 400) followed by the release of cargo (glucose and sodium carbonate); B,SEM micrograph of membrane surface of the osmotic pump before release; C,and after 24 hours of release experiment in M9 medium.

The semipermeable membranes of the control porosity osmotic pump tablet were observed by SEM before and after 24 hours of release study in an M9 medium. The micrographs displayed consistency with no defects on the surface of the membrane before the release study. At the end of the release, a porous membrane was observed due to leakage of the soluble pore-former component (PEG 400) of the coated membrane (Figure 2A and C).

### 3.2. In-vitro Drug Release of Osmotic Pump 

### 3.2.1 Glucose Release Profile from Osmotic Pumps

**Figure 3.**
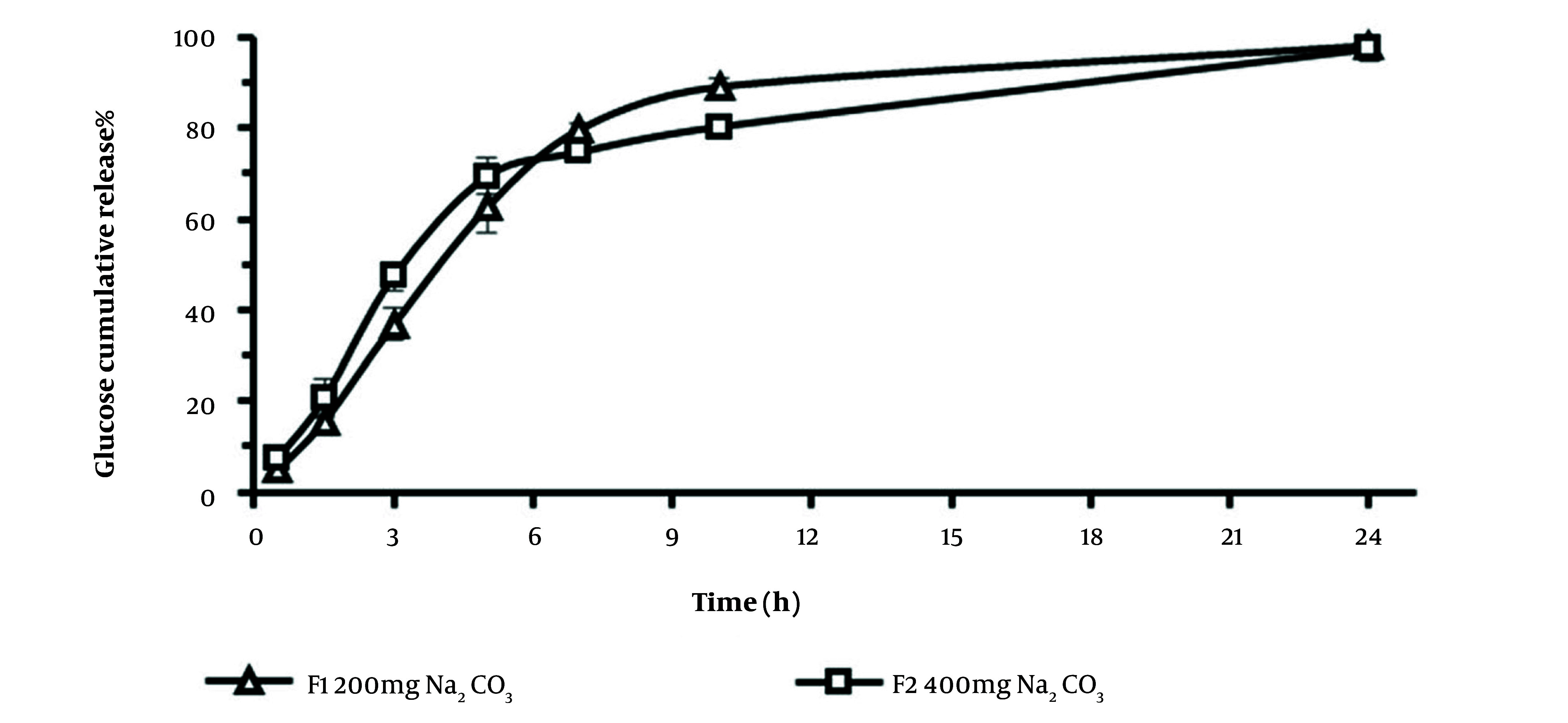
Cumulative glucose release profile (%) from osmotic pumps in M9 medium, from F1 and F2 formulations containing the same amount of glucose (1000mg) and different content of Na_2_CO_3,_ 200mg, and 400mg, respectively (mean ± SD, n = 3).

The cumulative release profiles of glucose from tablets with different Na_2_CO_3 _contents, 200 mg (F1), or 400 mg (F2) formulations were determined in the M9 medium for 24 hours (Figure 3). The calculated similarity factor value (*f*_*2*_ = 54) of the glucose release profiles was in the similarity range (> 50) according to Food and Drug Administration (FDA)-accepted criteria (30). Therefore, the release profile of glucose seemed to be independent of sodium carbonate content in the formulations. The cumulative amount of glucose released over 24 hours was similar in both systems. The slope of the release profile (release rate) for F2 is slightly higher than F1 (Table 2); nevertheless, the differences are not statistically significant. However, the amount of cumulative glucose released over 7 or 10 hours from F1 was significantly higher than those of F2 (*t*-test, P < 0.05), which might be due to the higher amount of MCC and its swelling properties (31) in the F1 core tablet that might lead to the faster leakage of the dissolved solution inside tablets.

#### 3.2.2. Na_2_CO_3_ Release Profile from Osmotic Pumps

**Figure 4.**
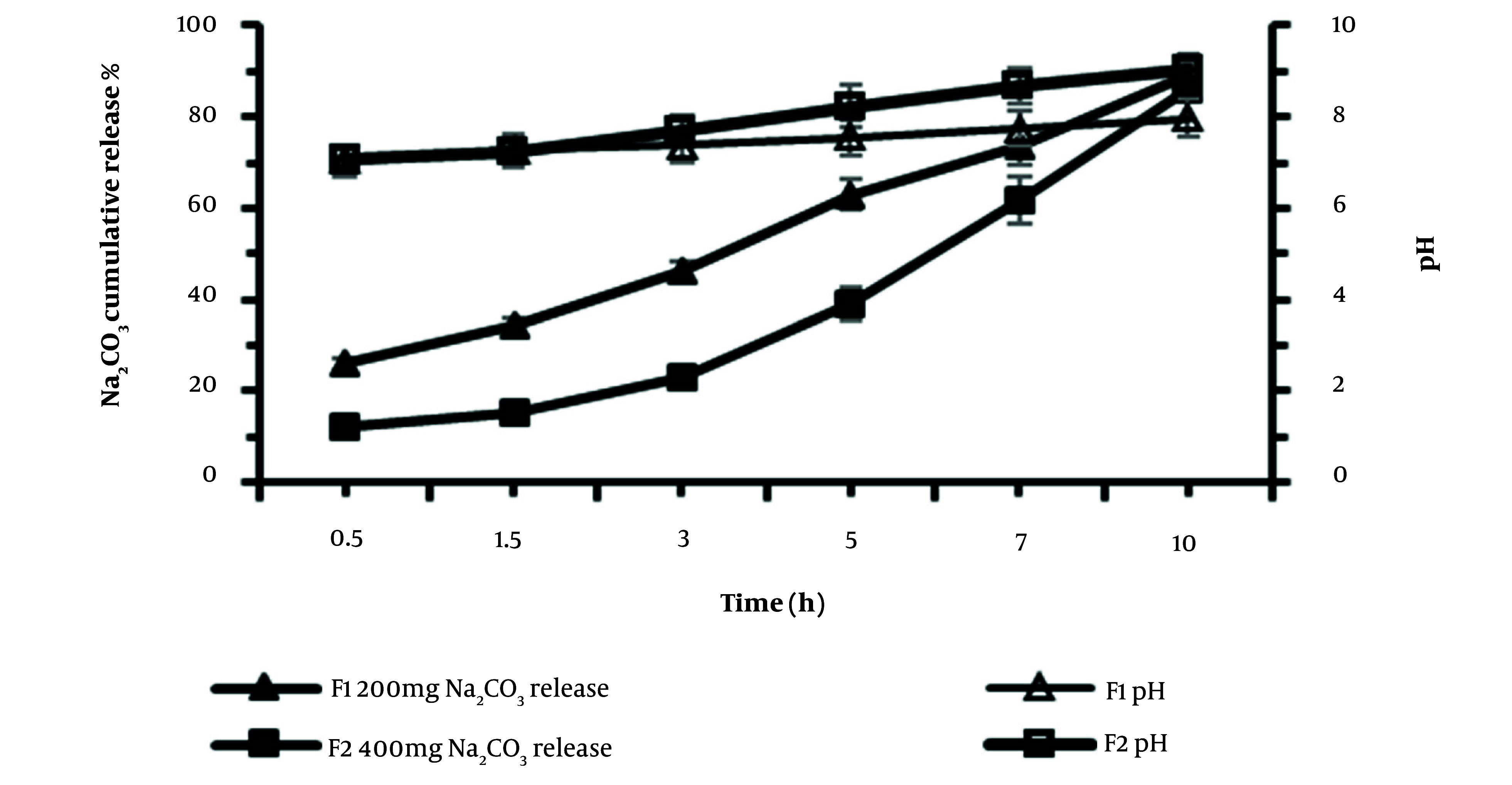
Cumulative Na_2_CO_3_ release profile % (closed symbols) and pH changes (open symbols) from osmotic pump tablets in M9 culture medium, from F1 (squares) and F2 (triangles) formulations containing the same amount of glucose (1000mg) and different content of Na_2_CO_3_, 200 mg, and 400 mg, respectively (mean ± SD, n = 3).

The release behavior of Na_2_CO_3_ from osmotic pump tablets with two different Na_2_CO_3_ contents, F1 (200 mg) and F2 (400 mg), was investigated. The data of the percentage cumulative Na_2_CO_3 _release and pH value are depicted in Figure 4. The results demonstrated that 89.8% and 86.2% of Na_2_CO_3 _of F1 and F2 formulations were released within 10 hours, respectively. The pH changes of the medium were higher in F2 formulation at sampling times 7 and 10 hours (*t*-test, P < 0.05). Up to 5 hours of release, F1 and F2 formulations showed no significant differences in increasing the pH of the medium; however, in the following hours, the higher Na_2_CO_3 _content of F2 led to higher pH. This can be considered an advantage of using an F2 formulation in bacterial growth culture since the pH reduction in the bacterial culture happens at the late hours of growth.

#### 3.2.3. Effect of Pore-Forming Level on the Release Profile

**Figure 5.**
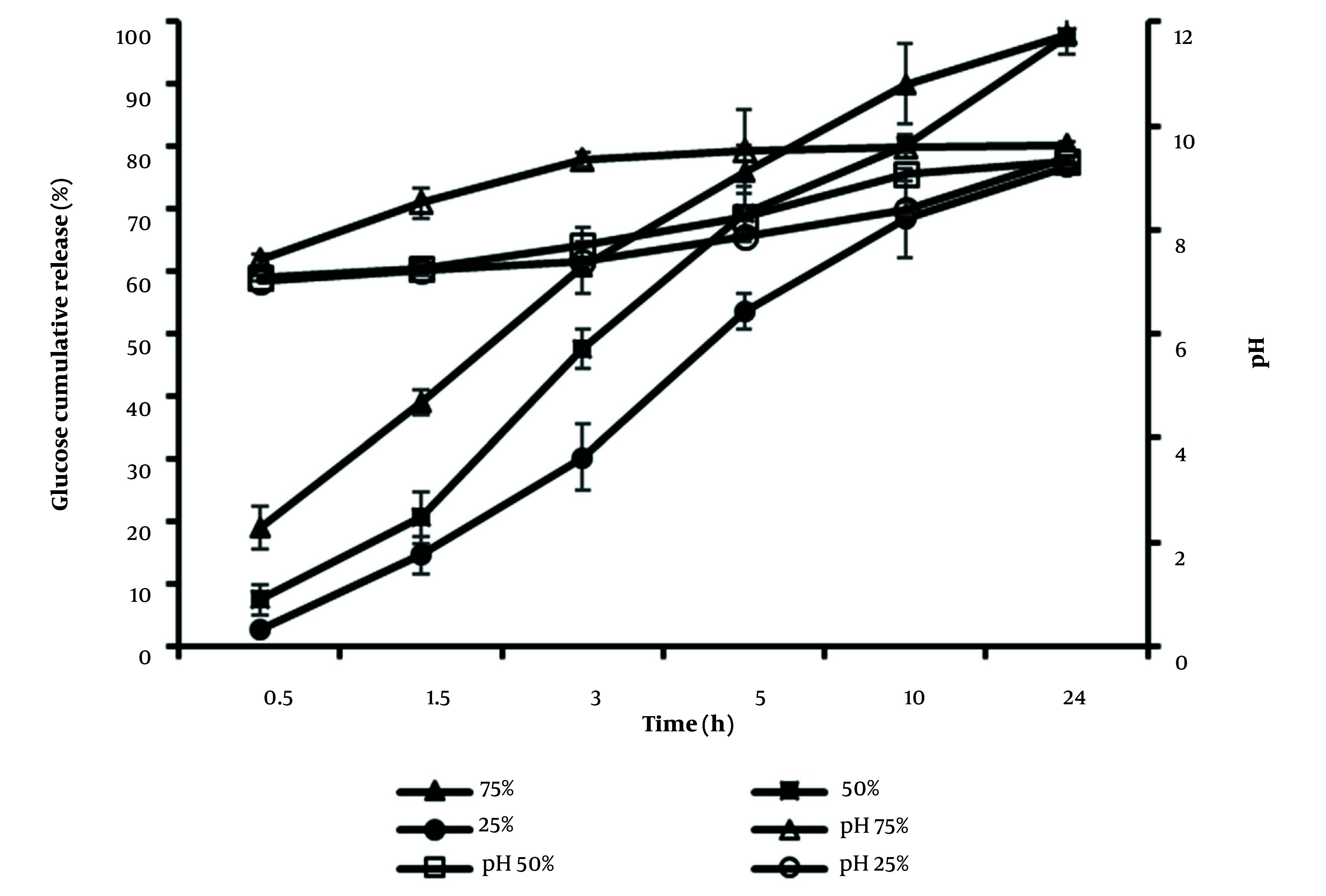
Effect of pore-forming (PEG 400) level, 25% (circles), 50% (squares), and 75% (triangles) in the coat solution of osmotic pumps on glucose release profile (closed symbols) and pH changes (open symbols) in M9 medium (mean ± SD, n = 3).

To investigate the effect of the pore-forming agent (PEG 400) content in the coat solution on the release, formulations with different percentages of PEG 400 (25%, 50%, and 75%) of CA (w/w) were prepared. The rate of release of formulations F3 (75%), F2 (50%), and F4 (25%) during 10 hours of release were 18.36, 17.52, and 14.57 % h^-1^. Therefore, a linear correlation was obtained between the drug release rate and the content of the pore former; consequently, a faster release rate was observed with a higher level of pore former (Figure 5). Furthermore, the similarity factor calculations between F2, F3 (45 < 50) and F2, F4 (43 < 50) confirmed the different release profiles of formulations. However, in agreement with bacterial growth conditions, there is no need for the high concentration of glucose and pH modifier agent at the first hours of growth; therefore, the formulation with a medium porous membrane (F2, 50%) was preferred, compared to the high (F3, 75%) and low (F4, 25%) porous membrane tablets. In addition, the release percentage of the F2 formulation up to 24 hours (97%) in the experiment was higher than that of F4 (76.71%).

#### 3.2.4. Kinetic of Drug Release

To determine the mechanism of glucose and Na_2_CO_3_ release from the optimized formulations (F1, F2), the release data were fitted to various mathematical models (zero-order, first-order, and Higuchi) using the best goodness-of-fit test (r^2^) (24). It is evident from the data (Table 2) that all CPOP formulations follow the zero-order model release as represented.

**Table 2.** Fitting of Release Data of Glucose and Na_2_CO_3_ of Optimized Formulations to Different Kinetic Models

Active Ingredients	Formulation Code	Zero-Order		First-Order		Higuchi	
		**K**_**0**_ ** (% h** ^**-1**^ **)**	**r** ^**2**^	**K**_**1**_ ** (h** ^**-1**^ **)**	**r** ^**2**^	**K**_**H**_ ** (% h** ^**-0.5**^ **)**	**r** ^**2**^
**Glucose**	F1	12.99	0.99	0.54	0.90	38.07	0.97
	F2	14.10	0.99	0.48	0.89	41.72	0.98
**Na**_**2**_ **CO**_**3**_	F1	7.98	0.99	0.19	0.97	26.32	0.98
	F2	8.95	0.99	0.26	0.97	29.73	0.94

### 3.3. Efficiency of the Osmotic Pump on E. coli Growth 

**Figure 6.**
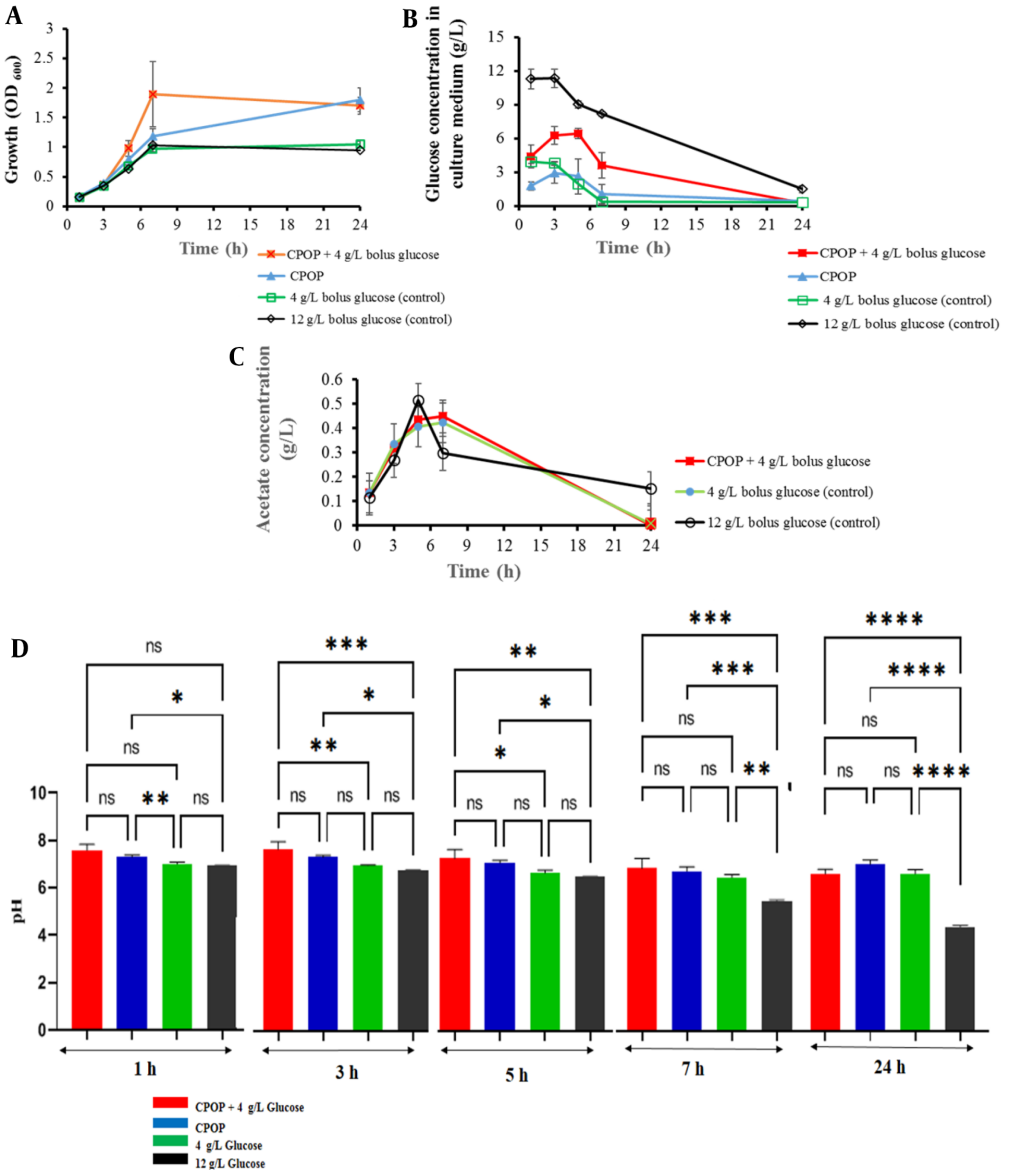
*Escherichia coli* (BL21) growth; A, glucose consumption; B, acetate production; C, and pH changes; D, in M9 cultures medium containing bolus glucose (4 g/L or 12 g/L) as controls in comparison to controlled delivery glucose by CPOP with 4 g/L bolus glucose or without bolus glucose (mean ± SD, n = 3). The data represent mean ± SD, n = 3. ns, P > 0.05. *, P ≤ 0.05. **, P ≤ 0.01. ***, P ≤ 0.001. ****, P ≤ 0.0001.

After optimizing the CPOP formulation, we compared the effect of the controlled release of glucose and Na_2_CO_3_ from the osmotic pump on the growth of *E. coli* to the one-step bolus feeding by glucose (4 g/L or 12 g/L) as control cultures. A sterile tablet (F2) was added to the *E. coli* culture in the M9 medium at the beginning of the bacterial growth. The effect of the tablet on bacterial growth was investigated with two approaches: The presence or absence of initial glucose (4 g/L) in the culture medium. Based on the glucose and Na_2_CO_3_ release profiles depicted in Figures 3 and 4, the contents of CPOP were released at a constant rate. The results of the experiments showed that applying the tablets had a significant influence on bacterial growth. After 7 hours in the log phase of *E. coli* growth, the culture containing an osmotic pump with the initial glucose had a higher bacterial density, and the mean OD_600_ was about 80% higher than that of cultures without tablets (1.9 *vs.* 1). In addition, at the end of the experiment, both conditions of added tablets (with or without 4 g/L glucose) had higher mass (1.5, 1.7 *vs.* 1) than cultures without tablets (Figure 6A). The results showed a significant improvement in the growth of bacteria at sampling times of 7 and 24 hours, based on the one-way ANOVA and least significant difference (LSD) post-hoc test (P < 0.05).

According to pH changes during cultivation, in the control culture containing no tablet (with 12 g/L initial glucose), pH decreased below 5.5 up to 7 hours (Figure 6D), which might be due to incomplete glucose consumption and acetate production (32). *Escherichia coli* grows aerobically; therefore, in the presence of an excess amount of glucose, the rate of glucose consumption increases, and respiration becomes a limiting factor. Due to the incomplete oxidation of glucose to CO_2_, the metabolites, such as acetate, are secreted to get rid of the extra redox potential (7, 33). The aforementioned findings urge the need to prepare controlled-release glucose systems to avoid its accumulation. The addition of a CPOP at the time of inoculation resulted in pH maintenance of about 6.8 in the sampling time of 7 hours (Figure 6D) due to the controlled release of glucose and Na_2_CO_3_. In the present investigation, the controlled release of glucose from the formulation led to a significant decrease in acetate production as a by-product in comparison to control cultures with no tablet (12 g/L bolus glucose), as depicted in Figure 6C. As a result, the utilization of continuous glucose and sodium carbonate release of the formulation led to elevating bacterial growth and pH maintenance in the desired range.

## 4. Discussion

Controlled delivery of nutrients and precise pH control is essential for optimal bacterial growth (34). Some studies on high cell density cultivations in shake flasks have focused on controlled-release feedings, such as using syringe pumps with controlled feed rates (35), slow-release systems in microtiter plates culture (36), or other controlled-release formulations (8, 10, 11). Osmotic pumps are considered promising controlled delivery systems due to their highly predictable and programmable release rate by adjusting the release parameters (16, 37). To the best of our knowledge, the osmotic pump tablets have not been used in bacterial culture. The osmotic pump was employed in this study for the delivery of glucose and sodium carbonate in *E. coli* cultivation in shake flasks to overcome the two main limitations of batch culture, such as the overflow metabolism of carbon sources and pH drift (38), while avoiding high buffer concentrations, any additional infrastructure like syringe pumps, and monitoring probes which has been reported for parallel nutrient feeding and pH monitoring in shake flasks in the former studies (39, 40).

To control the release rate of the osmotic pump, two strategies were employed in this study using a retardant agent in the core of the osmotic pump and the control of porosity on the semipermeable membrane by optimizing the concentration of pore-former in coat solution. To sustain the release rate of sodium carbonate and glucose from the osmotic pump during the first hours of bacterial growth, Eudragit L100, as a retardant agent (41), was used successfully in the core of the tablet. In addition, the content of the pore-forming agent (PEG 400) in the coating membrane was optimized due to its great effect on the release rate, as this technique was employed in some previous studies to regulate the release rate of metformin hydrochloride (42) and enalapril (43) from the osmotic pump. This advantage led to the use of CPOP at the beginning of growth so that the pH in the early stages of the growth period did not exceed 7.8, which correlates well with the optimal pH range (6.5 - 7.5) for *E. coli* growth (44). The excessive release rate of cargo, especially sodium carbonate, during the first hours of growth leads to high pH, which was reported in the previous studies with silicone polymer matrices (9). 

Moreover, the current study's designed CPOP was able to prevent pH from falling below 6.4 within 24 hours in the M9 culture medium. Sanil et al. also reported a hydrogel loaded with Mg(OH)_2_ capable of controlling the pH above 5.8 for up to 14 hours in an LB medium supplemented with glucose (11).

The production and use of CPOP is feasible and does not require a prior preparation and washing process to reduce toxicity (removal of unreacted monomer) or burst release reported with controlled release hydrogel disks that should be pre-incubated in water for 1 - 3 days before adding to the cell cultures (10, 11).

Moreover, the stability of the membrane of the osmotic pump in different media can be considered an advantage of using these systems, compared to enzyme-based controlled systems, which can be affected by enzymes and enzyme inhibitors (38). However, the timing of tablet addition and the number of tablets that can be added requires further evaluation since the osmotic pressure in the medium might affect the release of cargo from the osmotic pump, as reported in previous studies of osmotic pumps (17).

### 4.1. Conclusions

The controlled porosity osmotic pump was formulated with glucose (as carbon source feeding) and sodium carbonate (as pH modifier) and characterized regarding release behavior for optimum feeding and controlling pH in bacterial culture. Afterward, the influence of osmotic pump tablets containing glucose and sodium carbonate was evaluated in batch culture of *E. coli* (BL21). The controlled release of glucose from the controlled porosity osmotic pump, compared to bolus addition to culture medium in a flask, led to a decrease in overflow metabolism of glucose and a decrease of acetate secretion by *E. coli*. Furthermore, pH control by releasing sodium carbonate at a favorable rate from the tablet led to significantly higher biomass yield compared to blank with no tablet and containing bolus glucose. This finding highlights the utility of the osmotic pump for microbial shake flask cultures in fed-batch mode.

ijpr-23-1-138677-s001.pdf

## Data Availability

The data presented in this study are uploaded during submission as a part of the main manuscript file. Any further data and details are available for readers upon request.
