# Supplementary material for: Design and Characterization of an Osmotic Pump System for Optimal Feeding and pH Control in E. coli Culture to Increase Biomass
Source: Iran J Pharm Res. 2024 Feb 17;23(1):e138677. doi: 10.5812/ijpr-138677 (PMC11246646; doi:10.5812/ijpr-138677)
Supplement: ijpr-23-1-138677-s001.pdf [file ijpr-23-1-138677-s001.pdf]

**Appendix 1:**

**Culture medium preparation**: LB medium was prepared from yeast extract (0.5%), tryptone (1.0%), and NaCl (0.5%). The *E. coli* main cultivation for the experiment was performed in a mineral M9 medium that was purchased from Biobasic (Toronto, Canada). To prepare 1-liter culture media, 11.3 g M9 powder was used which is composed of $Na_2HPO_4$ (6.8 g), $KH_2PO_4$ (3.0 g), NaCl (0.5 g) and $NH_4Cl$ (1.0 g), then, supplemented with glucose (0.4% w/v), $MgSO_4$ (1 mM), $CaCl_2$ (0.3 mM), thiamin (1 mg/mL) and trace elements. The main cultures were inoculated with pre-culture (10:1) and the pH value of the medium was adjusted to 7.0 with HCl (1 M).

**Cell growth and pH determination**. cell growth was monitored by measuring the optical density at 600 nm ($OD_{600}$) of 1 mL samples at regular intervals. The samples were diluted with a fresh medium to measure the $OD_{600}$ in the linear range of the spectrophotometer (CT-1500-China).

One mL of the cell suspension was centrifuged (10000 rpm, 10 min) in 1.5 mL test tubes and the supernatant was used for the determination of glucose concentration, pH and acetate measurement. The pH measurements were made with a calibrated pH meter (744 Metrohm, Switzerland).

**Glucose assay**. Glucose was spectrometrically analyzed by the dinitrosalicylic acid (DNS) method according to previous studies (21,22) with minor modifications. Briefly, the DNS reagent and sample were mixed (2:1, v/v) in a 25 mL volumetric flask. Afterward, the flask was incubated in a boiling water bath for 5 min, cooled down in an ice bath, and then diluted to volume with deionized water. The absorbance was analyzed by UV-Vis spectroscopy (Cecil 2021 Milton, England) at 540 nm and used a calibration curve to determine glucose concentration.

**Sodium carbonate assay**. Sodium carbonate ($Na_2CO_3$) was assayed by using a calibration curve that was plotted based on measuring pH changes vs sodium carbonate concentration in an M9 medium. Sodium carbonate at concentrations of (0.5–4) mg/mL was used to plot the standard calibration curve. Each set of calibration data was plotted and linear regression was analyzed.

**Acetate concentration**. Acetate was measured by K-acetrm kit (Megazym, Ireland), according to the method described by the company in the manual that is based on acetate kinase (AK) and phosphotransacetylase (PTA). The absorbance was read by a microplate reader (BioTek Synergy HTX reader United states).
